# Extracellular Vesicles as Drivers of Lung Endothelial Dysfunction in ARDS: Mechanisms and Therapeutic Opportunities

**DOI:** 10.1002/cph4.70179

**Published:** 2026-06-04

**Authors:** Mohammed Yaman Al Matni, Patrick Belvitch, Steven M. Dudek, Eleftheria Letsiou

**Affiliations:** ^1^ Department of Internal Medicine Saint Louis University Saint Louis Missouri USA; ^2^ Division of Pulmonary, Critical Care, Sleep, and Allergy University of Illinois Chicago Chicago IL USA

## Abstract

Acute respiratory distress syndrome (ARDS) is a complex, life‐threatening condition that can arise from both direct pulmonary insults—such as pneumonia and aspiration—and indirect extrapulmonary causes including sepsis and trauma. A key driver of ARDS pathogenesis is the disruption of the lung endothelial barrier. Over the past several decades, extensive research has elucidated numerous mechanisms underlying the endothelial cell (EC) dysfunction in the acute lung injury (ALI) pathophysiology that occurs during ARDS. A range of EC modulators have been identified, as well as therapeutic strategies aimed at restoring EC integrity. More recently, extracellular vesicles (EVs) have emerged as critical mediators in ARDS pathogenesis, acting as potent regulators of endothelial function. EVs originating either locally in the lung or from distant tissues can reach the pulmonary vasculature, where they amplify inflammatory signaling and disrupt EC barrier integrity, thereby exacerbating injury and disease severity. This review summarizes recent advances in understanding how EVs from diverse cellular, tissue, and organ sources contribute to endothelial dysfunction in ALI pathophysiology. It also explores emerging endothelial‐targeted therapies for ARDS, spanning preclinical and clinical stages, that may counteract EV‐mediated pathogenic mechanisms, and highlights key gaps that remain to be addressed.

## Graphical abstract

This review summarizes how extracellular vesicles from diverse pulmonary and extrapulmonary sources contribute to endothelial dysfunction in ARDS and evaluates emerging endothelial‐targeted therapies for their potential to mitigate EV‐mediated pathogenic mechanisms.

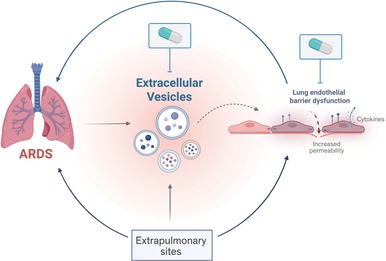

## 1 Introduction

Acute respiratory distress syndrome (ARDS) is a severe clinical condition marked by profound respiratory distress, widespread pulmonary inflammation, and reduced blood oxygen saturation (Matthay et al. 2019). First described in 1967 as a case series of 12 patients presenting with tachypnea, hypoxemia, and decreased lung compliance (Ashbaugh et al. 1967), ARDS has gained renewed attention in the wake of the global COVID‐19 pandemic (Zhou et al. 2020). Although pneumonia and sepsis are the most common causes, other triggers include aspiration of gastric contents, burns, trauma, and severe acute pancreatitis (Laake et al. 2022).

The pathogenesis of ARDS involves an acute lung injury (ALI) process that is primarily driven by disruption of the alveolar‐capillary barrier, excessive neutrophil infiltration, and the release of inflammatory mediators—processes that culminate in pulmonary edema (Meegan et al. 2024). Impaired alveolar fluid clearance further exacerbates fluid accumulation, disrupts gas exchange, and leads to hypoxemia (Matthay et al. 2019; Bos and Ware 2022). ARDS accounts for approximately 10.4% of all intensive care unit admissions and 23% of patients requiring mechanical ventilation (Bellani et al. 2016). Despite advances in supportive care, mortality remains high, ranging from 35% to 50% (Pham and Rubenfeld 2017). Survivors of ARDS often face long‐term complications, including physical limitations, cognitive impairments, and psychological disorders. Post‐ARDS interstitial lung fibrosis may persist for months, whereas prolonged immobility contributes to muscle weakness and deconditioning. Psychiatric conditions—such as depression, anxiety, and post‐traumatic stress disorder—are increasingly recognized, along with cognitive deficits (Mart and Ware 2020). Currently, supportive care, including lung‐protective ventilation and conservative fluid management, remains the cornerstone of ARDS treatment (Matthay et al. 2019). Although significant progress has been made in understanding the syndrome's ALI pathophysiology and studying potential pharmacologic interventions, no medical therapy has yet proven effective in reducing mortality. Thus, there remains an urgent and unmet need for effective strategies to treat or prevent ARDS.

The alveolar‐capillary barrier, composed of alveolar epithelial cells and microvascular endothelial cells (ECs), is the central site of injury in ARDS (Meegan et al. 2024). This barrier is primarily responsible for efficient gas exchange between the alveoli and the pulmonary circulation during respiration. Its disruption results in increased permeability to protein‐rich fluid and excessive recruitment of inflammatory cells, which represent the hallmarks of ALI pathophysiology. Understanding the mechanisms by which ALI‐relevant insults damage the alveolar‐capillary barrier is crucial for developing effective therapeutics. In this review, we specifically focus on the endothelial side of the barrier. Lung ECs have multiple functions, including the maintenance of vascular integrity, homeostasis, regulation of fluid exchange, and participation in immune responses (Vassiliou et al. 2020). Key features of lung endothelial dysfunction include inflammation, increased permeability, and cell death. During ALI, ECs are driven into a pro‐inflammatory state by external stimuli such as bacteria, viruses, or acid, as well as by internal mediators including reactive oxygen species (ROS), thrombin, and cytokines. This activation is marked by upregulation of adhesion molecules (VCAM‐1, ICAM‐1, and selectins), activation of NF‐κB signaling, oxidative stress, and enhanced secretion of pro‐inflammatory cytokines (e.g., IL‐6, IL‐8, IL‐1β) (Su et al. 2024). Although these mechanisms are part of the host defense, excessive production of inflammatory mediators, accumulation of immune cells, and activation of pro‐coagulant signaling further amplify inflammation and disrupt the endothelial barrier (Su et al. 2024). ALI insults also trigger signaling cascades that increase endothelial permeability through cytoskeletal reorganization and disruption of inter‐endothelial junctions (Su et al. 2024; Komarova et al. 2017). These complex processes result in barrier disruption, allowing leakage of fluid, proteins, and immune cells into the lung interstitial and alveolar space. In addition, during ALI, ECs undergo multiple forms of programmed and non‐programmed death, including necrosis, apoptosis, necroptosis, and pyroptosis, which further compromise barrier integrity and amplify inflammation (Su et al. 2024). Importantly, EC dysfunction can be driven by crosstalk with other cell types, including alveolar epithelial cells, immune cells, and circulating cells such as platelets and red blood cells (RBCs).

Extracellular vesicles (EVs) have emerged as important mediators of intercellular communication and inter‐organ crosstalk, and growing evidence implicates them in driving lung endothelial dysfunction during ALI. This review summarizes recent studies on the detrimental effects of EVs on endothelial function in ALI and highlights emerging endothelial‐targeted therapies for ARDS, with a focus on whether their protective effects may involve modulation of EV‐mediated signaling pathways.

## 2 EVs and Their Role in Endothelial Dysfunction in ALI/ARDS

EVs are lipid bilayer–delimited particles that are released by virtually all cell types under physiological conditions as well as during cellular activation or death (Mohammadipoor et al. 2023; Welsh et al. 2024). They represent a heterogeneous population that varies in size—from ~30 nm to several micrometers—along with differing in composition, mechanism of biogenesis, and function. EVs can be categorized into small EVs (< 200 nm) and large EVs (> 200 nm) (Welsh et al. 2024). Earlier studies classified EVs according to their biogenetic origin and size. In this framework, exosomes, which fall within the small EV category, were defined as vesicles released from intracellular compartments through the fusion of multivesicular bodies (MVBs) with the plasma membrane. In contrast, microvesicles—also referred to as ectosomes or microparticles and typically categorized as large EVs—were described as vesicles that bud directly from the plasma membrane. In addition, several other specialized terms have been used in the literature, such as apoptotic bodies, formed during apoptosis (Welsh et al. 2024). Since most current EV isolation methods do not selectively enrich EVs generated through distinct biogenetic pathways, it remains challenging to definitively identify EV subtypes on the basis of origin or size, particularly in the absence of universally accepted molecular markers that distinguish microvesicles, exosomes, or other EV categories. Consequently, studies referring to specific EV subtypes (e.g., exosomes) often describe heterogeneous EV populations rather than vesicles that can be reliably assigned to a particular category (Welsh et al. 2024). For this reason, the International Society for Extracellular Vesicles (ISEV) recommends using the generic term “EVs” unless a specific EV population has been rigorously isolated and thoroughly characterized (Welsh et al. 2024).

Independent of their classification, EVs are known to transport a diverse cargo of proteins, lipids, RNAs (including mRNA and miRNA), DNA, and even cellular organelles such as mitochondria. Extensive research in the past two decades has established that all types of EVs play a crucial role in mediating inter‐cellular as well as inter‐organ communication (van Niel et al. 2022). This occurs by the transfer of the EV molecular cargo to target cells in an autocrine, paracrine, or endocrine/exocrine manner. The EV cargo is determined by their originating cells and the conditions occurring during their generation. Multiple studies have demonstrated, for instance, that EVs derived from the same type of cells but activated by different stimuli possess unique cargo compositions (Letsiou and Bauer 2018; Letsiou et al. 2015). In addition, EV production levels, as well as EV uptake or interaction with other cells, are altered in response to various triggers (Soni et al. 2022). Thus, the biological impact of EVs is critically dictated by their molecular composition, abundance, and the specific mechanisms through which they engage and influence target cells.

A growing area of ARDS research focuses on the role of EVs in lung injury, with increasing interest in their impact on both disease progression and recovery (Letsiou and Bauer 2018; Bavuso et al. 2023; Hu et al. 2022; Lee et al. 2016; Lee, Abston, et al. 2018; Mahida et al. 2020). Experimental and clinical studies have shown that EV production is altered in ALI/ARDS and related conditions (e.g., sepsis, pneumonia, trauma) and that EVs from different cellular sources and tissues contribute to lung injury (Letsiou and Bauer 2018; Bavuso et al. 2023; Lee et al. 2016; Mahida et al. 2020). Given its strategic location and constant interaction with circulating cells, the lung endothelium is a prime target for EV‐mediated effects. Moreover, the lung endothelium may represent a downstream target for EVs originating in the alveolar space (Ghosh et al. 2017). In ALI, intra‐alveolar contents including inflammatory mediators (e.g., cytokines), surfactant proteins, and bacteria can translocate from the lungs into the bloodstream (Holmes et al. 2025; Uchida et al. 2006; Elmore et al. 2023). Lung‐to‐circulation transfer during ALI most commonly occurs as a consequence of impaired alveolar‐capillary barrier integrity and paracellular gap formation; however, additional pathways have also been identified. For example, neutrophils within the alveolar space have been described to re‐enter the bloodstream through a process known as reverse transmigration (Nourshargh et al. 2016). Although the mechanisms by which lung‐derived EVs access the circulation remain largely unknown, a growing body of literature describes multiple routes used by tissue‐derived EVs to enter the bloodstream. These include paracellular and transcellular transport across tissue barriers, as well as trafficking through the lymphatic system, as reviewed in (Iannotta et al. 2023).

**FIGURE 1:**
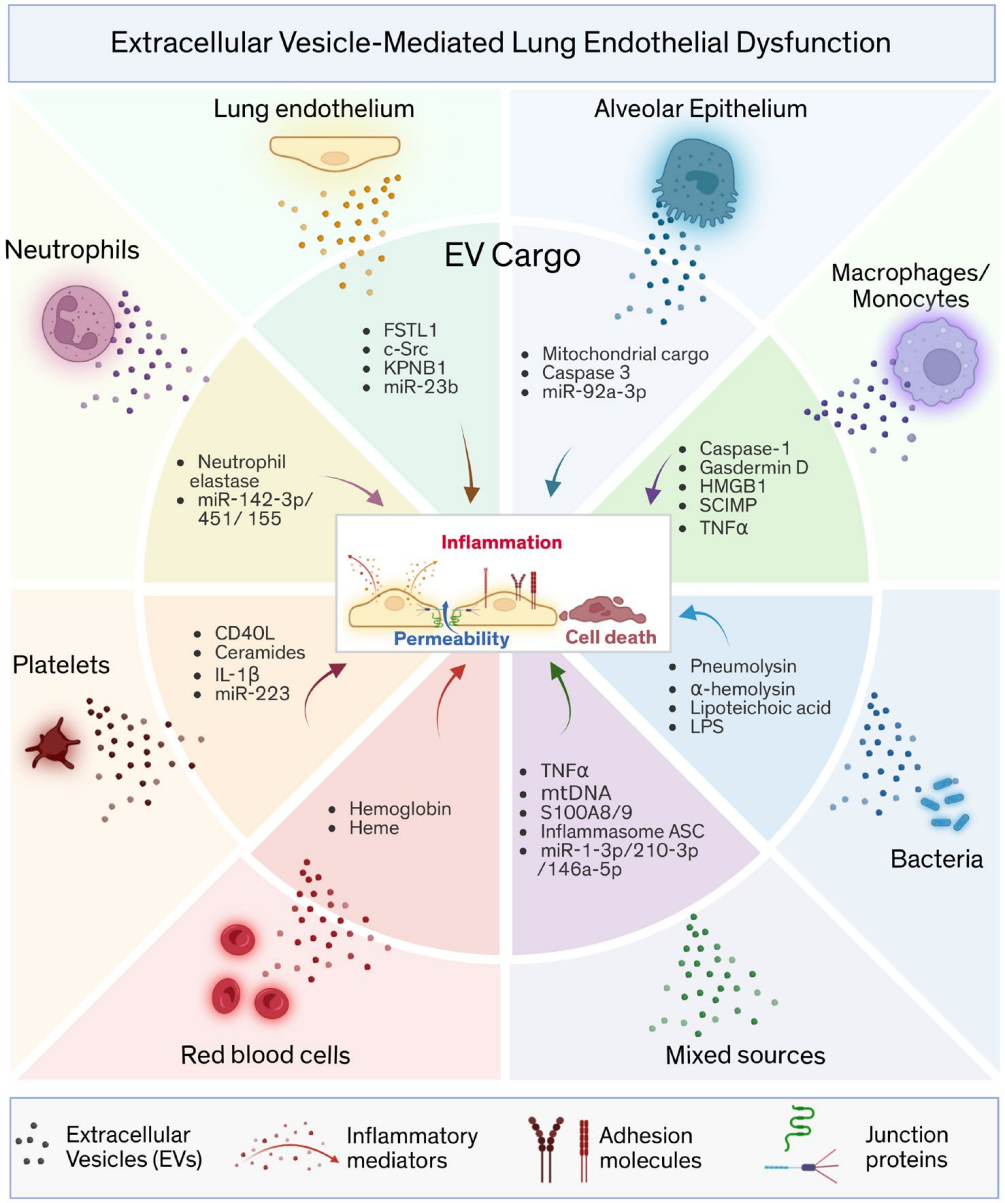
Extracellular vesicles from multiple sources may contribute to lung endothelial dysfunction in acute lung injury. Extracellular vesicles (EVs) are released by various cell types, including bacteria, during acute lung injury (ALI). Once released, these EVs interact with lung endothelial cells (ECs), triggering EC activation (e.g., upregulation of adhesion molecules), pro‐inflammatory signaling (e.g., secretion of cytokines), increased vascular permeability (e.g., disruption of intercellular junctions), and, in some cases, cell death. Representative EV‐associated molecules (EV cargo) that may impair EC function are shown for EVs originating from lung endothelial cells, alveolar epithelial cells, immune cells (macrophages, monocytes, neutrophils), platelets, red blood cells, and bacteria, as well as for EVs of mixed origin released into the circulation, the alveolar space, or from extrapulmonary organs. The figure was created with Biorender.com.

In this section, we review recent literature on pulmonary‐ and extrapulmonary‐derived EVs and their impact on endothelial barrier regulation in ALI (Figure 1). As discussed above and for consistency across studies, we use the generic term EVs to collectively refer to the vesicle populations described in the cited studies, without specifying individual EV subtypes (e.g., exosomes, microvesicles, small or large EVs).

### 2.1 Lung Endothelial‐Derived EVs

The lung vascular endothelium produces increasing levels of EVs with pro‐inflammatory properties in response to injurious stimuli, including bacterial products, mechanical stretch, and cytokines. EVs produced under these inflammatory conditions contribute to endothelial dysfunction as reviewed in detail in (Letsiou and Bauer 2018; Bavuso et al. 2023). More recent work has revealed specific mechanisms underlying these effects. For example, one study demonstrated that EC (HUVEC)‐derived EVs activate the TLR4/JAK3/STAT3/IRF‐1 signaling pathway in recipient ECs, promoting ALI through the delivery of follistatin‐like protein 1 (FSTL1) (Yuan et al. 2024). In another study, Chatterjee et al. reported that EC‐EVs produced by TNF‐α–stimulated HUVECs increased endothelial permeability by disrupting interendothelial junctions, an effect linked to their c‐Src cargo (Chatterjee et al. 2020). Similarly, EVs from lipopolysaccharide (LPS)‐treated ECs (isolated from pulmonary veins of rats) increase endothelial permeability in vitro and in vivo by transferring miR‐23b to the endothelium, where it targets ZO‐1 (Zheng et al. 2020). Pulmonary vascular leakage was reduced when animals received EVs from ECs pretreated with amitriptyline, an acid sphingomyelinase inhibitor that suppresses EV release (Zheng et al. 2020), highlighting the potential of EV‐targeted therapeutic strategies. Further, EC‐derived EVs may also contribute to endothelial barrier disruption and the pathogenesis of ALI through indirect mechanisms. For example, pulmonary ECs exposed to LPS release high levels of EVs enriched in karyopherin subunit beta‐1 (KPNB1) that stimulate neutrophils (PMNs) to upregulate neutrophil elastase (NE) (Zi et al. 2024). These activated PMNs undergo reverse transendothelial migration, a process that allows them to re‐enter the circulation with a highly inflammatory phenotype. Once in circulation, these PMNs exacerbate lung injury by degrading junctional adhesion molecule‐C (JAM‐C) at EC junctions via NE (Zi et al. 2024).

### 2.2 Lung Epithelial‐Derived EVs

The lung epithelium is a key site in ALI pathogenesis. Both infectious agents, such as bacteria and viruses (Letsiou, Teixeira Alves, et al. 2021; Soni et al. 2016; Zabrodskaya et al. 2022; Scheller et al. 2019), and non‐infectious stimuli, including hyperoxia and mechanical stretch (Moon et al. 2015; Wang, Xie, et al. 2022), can trigger the release of epithelial EVs with distinct molecular cargo. Several studies have explored the effects of epithelial EVs on immune regulation in ALI. Moon et al. demonstrated that during hyperoxia, lung epithelial EVs are enriched in caspase‐3 and activate inflammatory responses in macrophages through ROCK‐1 signaling (Moon et al. 2015). Upon LPS stimulation, EVs from the rat alveolar epithelial line RLE‐6TN carry miR‐92a‐3p and activate NF‐kB signaling in macrophages in vitro and in vivo, contributing to ALI (Liu et al. 2021). Because both NF‐kB and ROCK‐1 signaling pathways are known drivers of EC dysfunction, epithelial‐derived EVs carrying caspase‐3 or miR‐92a may represent an additional mechanism contributing to EC injury. Further, our work has shown that exposure of alveolar epithelial cells to pneumolysin, a potent virulence factor of *Streptococcus pneumoniae*, induces the release of EVs enriched with inflammatory mitochondrial components (Letsiou, Teixeira Alves, et al. 2021; Nerlich et al. 2018). These epithelial EVs are readily taken up by neutrophils, modulating their functional responses (Letsiou, Teixeira Alves, et al. 2021). Notably, our ongoing studies suggest that these EVs also target the pulmonary endothelium, promoting increased vascular permeability and inflammation (unpublished data) (Letsiou et al. 2024; Letsiou et al. 2023). Consistent with these observations, other studies have demonstrated that EVs from septic patients, LPS‐stimulated monocytes, or TNF‐α‐treated HUVECs carry mitochondrial cargo that activates ECs and induces pro‐inflammatory signaling (Zhang et al. 2023; Puhm et al. 2019; Manzar et al. 2025; Peng et al. 2026). Taken together, these studies support the concept that EVs enriched in mitochondrial components, derived from various sources, including alveolar epithelial cells, may serve as important mediators of lung endothelial inflammation and vascular dysfunction. Despite these findings, the impact of epithelial‐derived EVs on pulmonary endothelial functions remains largely unexplored, and further studies are needed to address this knowledge gap.

### 2.3 Immune Cell‐Derived EVs

Monocytes are a significant source of EVs that can cause lung EC dysfunction. Studies by Mitra et al. have demonstrated that THP‐1 cells, a human monocyte cell line, treated with LPS release EVs carrying active caspase‐1 and gasdermin D, which are taken up by lung microvascular EC, leading to their apoptosis (Mitra et al. 2015; Mitra et al. 2018). Importantly, gasdermin‐containing EVs are increased in septic patients with ARDS (Mitra et al. 2018), providing strong evidence that these EVs could represent critical mediators of monocyte‐endothelial communication contributing to ARDS pathogenesis.

Previous work by Karpurapu et al. emphasized the importance of macrophage‐EC crosstalk in ALI pathogenesis. Co‐culture experiments revealed that LPS‐activated macrophages increase endothelial permeability (Karpurapu et al. 2018). Notably, this effect is dependent on NFATc3 (Nuclear Factor of Activated T‐cells 3) signaling, as pulmonary microvascular ECs co‐cultured with NFATc3‐deficient macrophages exhibited reduced permeability (Karpurapu et al. 2018). Pharmacological inhibition of NFATc3 using a novel cell‐permeable calcineurin peptide (CNI103) attenuated pulmonary edema and ALI in LPS‐treated mice (Karpurapu et al. 2018). Follow‐up studies demonstrated that EVs isolated from bronchoalveolar lavage (BAL) fluid of LPS‐treated mice were enriched in arachidonic acid (AA) metabolites and disrupted EC barrier integrity. In contrast, BAL EVs from CNI103‐treated mice contained lower levels of AA‐derived lipids and were less disruptive to the EC barrier (Karpurapu et al. 2024). These findings implicate NFATc3 in EC barrier dysfunction via AA‐enriched EVs. However, given the heterogeneous cellular origin of BAL EVs, the specific contribution of macrophage‐derived EVs carrying AA lipids to EC permeability remains to be clarified. Another recent study demonstrated that lactate, which is elevated during sepsis, activates macrophages to release HMGB1 within EVs; these EVs induce EC activation and junctional disruption in HUVEC (Yang et al. 2021), highlighting a potential mechanism by which sepsis causes ARDS.

Other recent studies have shown that macrophages release EVs containing SCIMP (SLP65/Csk‐interacting membrane protein), which acts as a chemoattractant for peripheral neutrophils in *E. coli* pneumonia‐induced ALI models (Pei et al. 2024). Given SCIMP's role as a scaffold for the Src family kinase Lyn in activating TLR4 signaling (Luo et al. 2020), it will be helpful to determine how SCIMP‐enriched macrophage‐derived EVs influence lung EC function. In addition, alveolar macrophages infected with methicillin‐resistant *Staphylococcus aureus* (MRSA) release EVs that promote vascular permeability and inflammation in vivo (Bai et al. 2024). Mechanistically, EVs derived from MRSA‐treated macrophages exhibit distinct proteomic and miRNA profiles compared with controls. These MRSA‐induced EVs trigger necroptosis in recipient macrophages through the delivery of TNF‐α and miR‐146a‐5p (Bai et al. 2024). Although it remains unknown whether these EVs directly target the lung endothelium, this possibility represents an intriguing avenue for future investigation.

In ARDS, neutrophils are rapidly activated and recruited to the lungs. Excessive neutrophil activation leads to the release of potent inflammatory mediators, which significantly exacerbate lung injury and contribute to disease severity (Matthay et al. 2019). Upon activation, neutrophils release EVs, yet the roles of neutrophil‐derived EVs (NDEVs) in disease remain debated. Reports describe both pro‐ and anti‐inflammatory actions (Zhou and Bréchard 2022; Esquivel‐Ruiz et al. 2021), and, correspondingly, both barrier‐disruptive and barrier‐protective effects on endothelium, as summarized in (Ma et al. 2019; Kolonics et al. 2020). A recent study emphasizing the pro‐inflammatory arm showed that activation of neutrophils with fMLP, LPS, or PMA produces EVs enriched in miRNAs (miR‐142‐3p, miR‐451) that transfer to dermal microvascular ECs, triggering EC apoptosis and the release of pro‐inflammatory cytokines (Glémain et al. 2022). In another notable example, NDEVs delivered miR‐155 to human coronary arterial ECs, activating NF‐κB and eliciting additional injurious endothelial responses (Gomez et al. 2020). The role of NDEVs in ALI, particularly their interactions with the lung endothelium, remains largely unexplored. NDEVs have been reported to pack enzymatically active neutrophil elastase (NE). These NE‐associated EVs associate with collagen fibrils via Mac‐1 and degrade the extracellular matrix (Genschmer et al. 2019). Since NE plays an important role in endothelial glycocalyx degradation and permeability (Ushakumari et al. 2022; Suzuki et al. 2019), EVs carrying NE could represent critical mediators of lung endothelial barrier failure in ALI.

### 2.4 Platelet‐Derived EVs

Multiple lines of evidence demonstrate that activated platelets release high amounts of EVs with pro‐inflammatory and pro‐coagulant properties (Muttiah et al. 2024). Platelet‐derived EVs in stored blood products contribute to the pathogenesis of transfusion‐related ALI (TRALI) by targeting the endothelium (Kuebler et al. 2023). Apheresis platelet concentrate contains EVs that carry CD40L and induce lung microvascular EC damage in the presence of neutrophils (Xie et al. 2015). McVey et al. further demonstrated that platelets stored over time (5 days versus 1 day) release increased amounts of EVs, which are enriched in long‐chain ceramides and depleted of S1P (McVey et al. 2021). Importantly, platelet‐derived EVs collected at day 5 induce endothelial barrier disruption both in vitro and in vivo (McVey et al. 2021), providing compelling evidence of their detrimental interaction with the endothelium in driving lung injury. Numerous other studies have shown that platelet EVs can affect endothelial functions in inflammatory conditions. Thrombin‐activated platelets release microRNA‐223‐containing EVs, which can directly induce apoptosis in HUVECs (Pan et al. 2014). LPS‐triggered platelet EVs cause endothelial barrier disruption and upregulate inflammatory cytokines and adhesion molecules via phosphatidylserine binding, responses that can be eliminated in the presence of annexin 5 (Tschirhart et al. 2023). Similarly, EVs derived from thrombin‐activated platelets increased angiopoietin‐2 and endocan levels in septic animals, indicating enhanced activation and dysfunction of the endothelium (Jiang et al. 2023). Finally, during dengue infection, platelet activation triggers the release of EVs, rich in IL‐1β, which enhance endothelial permeability and stimulate ECs to release pro‐inflammatory markers (Hottz et al. 2013; Vedpathak et al. 2023). Despite these studies, further research is essential to elucidate the specific roles of platelet‐derived EVs in regulating lung endothelial function within the context of ALI.

### 2.5 RBC‐Derived EVs

RBC transfusion has also been implicated in the pathogenesis of TRALI, with growing evidence highlighting EVs as key mediators of the associated inflammatory response (Kuebler et al. 2023). Similar to stored platelets, packed RBCs (pRBCs) release EVs, which activate lung ECs, leading to the release of pro‐inflammatory cytokines, such as IL‐6 and KC (mouse homologue of IL‐8), and promoting the shedding of adhesion molecules, including VCAM‐1 and ICAM‐1 (Chang et al. 2017; Wattley et al. 2024). Additionally, EVs derived from stored pRBCs stimulate the release of P‐selectin and von Willebrand factor (vWF) from lung EC via a protein kinase C (PKC)‐dependent pathway (Sisak et al. 2024). These EVs cause lung EC activation upon their endocytosis, a process regulated by Rab5, a small GTPase protein (Kim, Abplanalp, et al. 2018). RBCs are also known to respond to various ALI‐relevant stimuli and contribute to the pathogenesis of ARDS by releasing cell‐free hemoglobin (CFH) (Janz and Ware 2015). CFH and metabolites, such as heme and hemin, not only have been well‐characterized for contributing to endothelial activation and hyperpermeability (Tomasek et al. 2021; Singla et al. 2017; James et al. 2020; Conger et al. 2023), but also have been recognized as EV‐associated molecules (Pat et al. 2022; Camus et al. 2015). These findings suggest that RBC‐EVs generated during ARDS may play an important role in regulating pulmonary endothelial function, warranting further investigations.

### 2.6 EVs Within the Alveolar Space

In ARDS, the alveolar space becomes a rich reservoir of EVs originating from various cell types, including macrophages, neutrophils, monocytes, platelets, epithelial and ECs (Lee et al. 2016; Letsiou, Teixeira Alves, et al. 2021; Soni et al. 2016; Costantini et al. 2024; Letsiou, Alves, et al. 2021; Mahida et al. 2022; Zareba et al. 2021). These alveolar EVs are potentially critical modulators of inflammation and vascular dysfunction, transporting bioactive molecules and altering intercellular communication (Soni et al. 2022; Zareba et al. 2021). Numerous studies, as reviewed in (Zareba et al. 2021; Hwang et al. 2022; Liu et al. 2022), have explored the content of BAL‐EVs in ALI and related conditions, such as pneumonia, ventilator‐induced lung injury (VILI), and sepsis. These EVs carry a variety of inflammatory mediators and miRNAs targeting downstream cells, influencing the severity of lung injury. For example, BAL‐EVs from LPS‐treated mice carry TNF‐α and induce inflammatory signaling in alveolar epithelial cells (Soni et al. 2016). Another study showed that secretory phospholipase A2‐IIA (sPLA2‐IIA), an inflammatory enzyme involved in AA production and vascular inflammation, was associated with EVs in patients with ARDS (Papadopoulos et al. 2020). There is also evidence suggesting that alveolar‐derived EVs may adversely affect the pulmonary vasculature; as mentioned above, EVs released into the alveolar space in mice treated with intranasal LPS contained high amounts of AA metabolites (prostanoids and HETEs), which can directly cause endothelial barrier disruption (Karpurapu et al. 2024).

### 2.7 Circulating EVs

ARDS is a complex syndrome that may arise from direct or pulmonary causes, such as severe pneumonia or aspiration, as well as from indirect or extrapulmonary causes, such as brain injury or acute pancreatitis. Sepsis, the most common cause of ARDS, can originate from infections in the lungs or from other organ systems (Li, Lin, et al. 2025). Therefore, in ARDS, the lung endothelium can be targeted by inflammatory mediators, including EVs, originating from the lung or reaching it via the circulation from extrapulmonary organs.

Numerous studies have evaluated the functional roles of circulating EVs isolated from plasma/serum of patients or animals with ARDS/ALI or sepsis, as reviewed in detail elsewhere (Bavuso et al. 2023; Mahida et al. 2020; Esquivel‐Ruiz et al. 2021; You et al. 2025; Lanyu and Feilong 2019). These studies contribute to our understanding of how mixed‐source EVs produced in vivo participate in ALI. For example, in sepsis‐induced ARDS, plasma EVs from septic patients increase EC permeability by delivering miR‐210‐3p. This specific miRNA targets ATG7 expression to cause inflammation and autophagy dysregulation in lung ECs (Li, Wang, et al. 2021). These findings were reproduced using the cecal ligation and puncture (CLP) model, an experimental sepsis‐related ALI (Li, Wang, et al. 2021). This model exemplifies interorgan communication, where systemic inflammation originating in the abdomen affects the lung endothelium. Another study showed that EVs from CLP‐treated rats carry increased levels of miR‐1‐3p (Gao et al. 2021). miR‐1‐3p targets and suppresses SERP1, leading to EC apoptosis, inflammation, and increased permeability (Gao et al. 2021). Distinct differences in the miRNA cargo of circulating EVs between septic patients with and without ARDS were recently revealed (do Nascimento et al. 2025). Given the important role of EV‐associated miRNAs in modulating EC function during ARDS, it is interesting to speculate that EVs from septic patients with ARDS exert a greater disruptive effect on the endothelial barrier compared to those from patients without ARDS. Besides miRNA, circulating EVs carry a variety of molecules that can damage the endothelial barrier. Plasma from patients with sepsis or septic shock contained significantly higher levels of S100A8/A9‐associated EVs in those who developed ARDS compared to those without ARDS (Wang, Wu, et al. 2025). These EVs have been shown to induce ALI by activating macrophages, whereas other studies have demonstrated that EV‐associated S100A9 directly promotes lung microvascular endothelial hyperpermeability (Wang et al. 2038). Another recent study demonstrated that EVs from sepsis patients carry mtDNA and trigger endothelial mitochondrial dysfunction and barrier disruption via PKCδ, promoting sepsis progression (Peng et al. 2026).

Beyond sepsis, in indirect ARDS, EVs released from extrapulmonary organs may contribute to lung endothelial injury. In patients with severe brain traumatic injury (TBI), serum EVs contain increased levels of the inflammasome protein ASC compared to those obtained from controls (Kerr et al. 2019). These EVs trigger pyroptosis in human lung ECs, suggesting that this pathway may contribute to TBI‐induced ARDS and highlighting EVs as potential mediators of brain–lung communication (Zhang et al. 2021). Patients with acute pancreatitis are at high risk for developing ARDS. Circulating EVs from a mouse model of severe acute pancreatitis express integrins through which they interact with the lung endothelium to cause lung EC junctional disruption and trigger pro‐inflammatory signaling (Hu et al. 2023). Transfusion‐related ALI represents another example of an indirect cause of ARDS, and as reviewed above, EVs play a major role in mediating the inflammatory and endothelial injury responses that drive its pathogenesis.

Circulating EVs may also arise from systemic conditions, such as obesity, a risk factor for ARDS. Zhang et al. found that EVs from obese patients exacerbated lung injury in mice subjected to high tidal volume mechanical ventilation (VILI). These EVs (from obese patients) carry reduced levels of miR‐150‐5p compared to normal controls. Reduced levels of miR‐150‐5p were linked to upregulation of XBP1 and RAB7, which promote VE‐cadherin endocytosis and lysosomal degradation, ultimately leading to increased endothelial permeability (Zhang, Gu, et al. 2025).

### 2.8 Bacterial EVs

EVs are key mediators not only of intercellular and interorgan communication, but also of interspecies interactions, particularly in the context of host–pathogen dynamics. Similar to eukaryotic cells, both gram‐positive and gram‐negative bacteria release EVs with potent pro‐inflammatory properties (White et al. 2021). EVs derived from *Pseudomonas aeruginosa* (PA) cause barrier disruption in lung microvascular EC in vitro and induce lung injury in mice primed with a low dose of LPS (McVey et al. 2022). Although the precise mechanisms by which PA‐EVs exert their injurious effects remain unclear, other bacterial EVs have been more thoroughly characterized. For instance, *Escherichia coli* EVs are internalized by microvascular ECs, where they upregulate adhesion molecules such as ICAM‐1, VCAM‐1, and E‐selectin, and stimulate robust IL‐8 production via NF‐κB and TLR4‐dependent pathways (Lee, Yoon, et al. 2018; Kim et al. 2013). Additionally, EVs from *Klebsiella pneumoniae* accelerate EC senescence and promote superoxide anion generation by downregulating SIRT1, further contributing to endothelial dysfunction (Li, Cui, et al. 2025). Although these studies did not identify the specific EV‐associated cargo responsible for the observed downstream effects, other research has demonstrated that EVs derived from pathogenic bacteria transport virulence factors. These include toxins such as pneumolysin (Codemo et al. 2018) and α‐hemolysin (Thay et al. 2013), as well as components like LPS and lipoteichoic acid (Krause et al. 2025; Kadurugamuwa and Beveridge 1995), all of which are well‐established drivers of EC injury (Letsiou et al. 2015; Gutbier, Schönrock, et al. 2018; Wang, Letsiou, et al. 2022; Pai et al. 2012).

In summary, despite differences in cellular origin and cargo composition, EVs derived from pulmonary and extrapulmonary cells, as well as from bacterial sources, converge on a limited set of shared endothelial injury pathways. Across EV sources, common effects include disruption of endothelial junctions, extracellular matrix degradation, upregulation of adhesion molecules (e.g., VCAM1), and enhanced release of inflammatory mediators, as well as induction of EC death (Figure 1). Collectively, these processes promote increased endothelial permeability and sustained propagation of vascular inflammation. This mechanistic convergence supports the concept that diverse EV populations function as coordinated amplifiers of endothelial barrier dysfunction in ARDS.

#### 2.8.1 Limitations and Considerations

It is important to acknowledge key considerations and limitations within the EV field and in the present review. First, we have not systematically evaluated the extent to which individual studies employed rigorous validation strategies to confirm EV–specific effects. The majority of evidence regarding the effects of EVs on endothelial function is derived from in vitro studies. In these studies, EVs are typically isolated from cells exposed to specific stimuli and subsequently applied to EC. Although this approach is widely accepted, it presents challenges in the context of EV research. Specifically, residual stimuli used to generate EVs may co‐isolate with EV preparations, potentially confounding experimental outcomes. As a result, the observed endothelial dysfunction may be attributable, at least in part, to contaminating stimuli rather than to EV‐mediated effects per se. To address this concern, investigators have implemented a range of validation approaches, including assessing EV activity under experimental conditions that minimize or eliminate the biological effects of the original stimuli. For example, LPS, a commonly used trigger of EV release, requires the presence of serum‐derived soluble CD14 (cluster of differentiation 14) to induce downstream signaling in EC. Accordingly, EVs isolated from LPS‐treated cells can be functionally evaluated in serum‐free conditions to distinguish EV‐specific effects from LPS carryover. Using this approach, Puhm et al. demonstrated that EVs derived from LPS‐treated monocytes activated ECs in the absence of serum (Puhm et al. 2019). Other validation strategies include assessing the functional relevance of key EV‐associated components. For example, Glémain et al. reported that EVs derived from TNF‐α–treated neutrophils induce EC inflammation (Glémain et al. 2022). In this study, the authors identified specific EV cargo elements that contribute to this response. Notably, they demonstrated that selected EV‐enriched microRNAs were sufficient to recapitulate the pro‐inflammatory effects of EVs on EC, thereby supporting a causal role for EV‐associated miRNAs in mediating endothelial activation (Glémain et al. 2022). In another example, EVs derived from TNF‐α–treated EC carry the kinase c‐Src and disrupt endothelial barrier integrity (Chatterjee et al. 2020). Silencing c‐Src in the parent cells prior to TNF‐α stimulation resulted in the release of EVs with significantly attenuated barrier‐disruptive effects. In both studies, the authors provided indirect evidence that specific EV cargo, rather than contaminating soluble factors or residual stimuli, is responsible for the observed endothelial responses.

Another key consideration is the heterogeneous nature of EVs and the variability introduced by different isolation protocols used across studies. As discussed above, EVs comprise several subtypes that are broadly classified on the basis of characteristics such as size and biogenesis pathway. In most experimental settings, investigators select an isolation method with the aim of enriching for a particular EV subtype (e.g., exosomes versus ectosomes, or small versus large EVs). However, no currently available isolation approach can reliably produce a pure population of a single EV subtype, as EVs overlap in size and lack definitive, subtype‐specific markers (Welsh et al. 2024). Consequently, EV preparations obtained using standard isolation methods are inherently heterogeneous. On the basis of these considerations, in this review, we use the overarching term “EVs” to encompass studies that may include mixtures of small and/or large vesicles. Although this inclusive approach allows for broader comparison across literature, it may obscure subtype‐specific effects and potentially overlook important differences in how distinct EV populations influence EC function.

It is also important to note that the studies discussed in this section examine EV effects across multiple EC types. ECs are not a uniform population but instead display substantial heterogeneity, with differences in structure, gene expression, junctional organization, and function depending on organ, vascular bed, and microenvironment. In the lung, macrovascular and microvascular lung ECs share core endothelial features and responses to inflammatory stimuli; however, pulmonary microvascular ECs possess a uniquely specialized and tightly regulated barrier optimized for gas exchange and fluid homeostasis (Meegan et al. 2024; Stevens 2011; Rizzo et al. 2020; Al Matni et al. 2024). Moreover, the lung microvasculature—the primary site of barrier failure in ARDS—contains distinct EC subtypes, including aerocytes and general capillary ECs, each with specialized roles. In addition, many in vitro studies use commercially available or primary ECs isolated from lung tissues, which often represent heterogeneous or mixed populations. For example, commercially available human lung microvascular ECs are often a mixture of both blood vascular and lymphatic ECs. Although such limitations are common in vascular biology, endothelial heterogeneity and cell purity may influence EV uptake and downstream signaling. Consequently, EV‐mediated effects observed in commonly used macrovascular models, such as HUVECs, may not fully recapitulate lung microvascular responses. Future studies dissecting EV signaling across defined endothelial subtypes will be important for understanding vascular vulnerability and therapeutic targeting.

In summary, it is important when interpreting EV‐related findings to carefully consider the experimental design, including the isolation protocol of EVs, the stimuli used to induce their release, the target cells, and the validation strategies employed to distinguish EV‐mediated effects from potential confounding factors.

## 3 Emerging Therapeutics That Protect the Endothelial Barrier in ARDS

Given the central role of endothelial dysfunction in the pathogenesis of ARDS, the development of endothelial‐targeted therapeutics represents a promising strategy to improve outcomes in this high‐mortality condition. In this section, we will review emerging therapeutics that are under clinical (Table 1) or pre‐clinical development and discuss their potential role in modulating EV‐mediated responses (Table 2). As discussed in the preceding section, injurious EVs contribute to ARDS pathogenesis by transporting inflammatory cargo (cytokines, miRNAs, etc.), activating recipient cells, and propagating inflammation. Accordingly, evaluating whether and how emerging therapeutics may directly or indirectly target EV production, cargo, or downstream signaling is of particular interest. We further highlight key knowledge gaps and propose conceptual frameworks to guide future investigation in this evolving area.

**TABLE 1:** Clinical trials in ARDS: Emerging therapeutics with endothelial protective properties.

Therapeutic agent	Mechanism of action	Patients recruited	NCT no.	Sponsor/phase
ALT‐100 mAb	Anti‐extracellular NAMPT antibody	Moderate–severe ARDS	NCT05938036	Aqualung Therapeutics Corp./Phase 2a
Paridiprubart	Anti‐TLR4 monoclonal antibody	ARDS	NCT06701669	PPD Development, LP/Phase 2
Vilobelimab	Anti‐C5a monoclonal antibody (complement pathway inhibitor)	ARDS	NCT06701682	PPD Development, LP/Phase 2
STSA‐1002	Anti‐C5a monoclonal antibody (complement pathway inhibitor)	ARDS	NCT07208591	Staidson (Beijing) Biopharmaceuticals Co. Ltd./Phase 3
Bevacizumab	Anti‐VEGF monoclonal antibody	ARDS	NCT06701656	PPD Development, LP/Phase 2
Fostamatinib	SYK kinase inhibitor	ARDS	NCT06564207	Inova Health Care Services/Phase 2
GEn‐1124	p38α:MK2 Dual signal modulator	ARDS	NCT05795465	Gen1E Lifesciences/Phase 2
Sivelestat	Neutrophil elastase inhibitor	Sepsis	NCT04973670	Southeast University, China/Phase 3
Sivelestat	Neutrophil elastase inhibitor	Moderate–severe ARDS	NCT06387823	Peking Union Medical College Hospital/Pilot
Anakinra	IL‐1 receptor antagonist	Moderate–severe ARDS	NCT05914454	Azienda Sanitaria‐Universitaria Integrata di Udine/Phase 2
AV‐001	Tie2‐activating PEGylated peptide	Pneumonia‐associated ARDS	NCT05123755	Vasomune Therapeutics Inc./Phase 2a
IC14 (Atibuclimab)	Anti‐CD14 monoclonal antibody	Mechanically ventilated ARDS	NCT06513949	Implicit Bioscience/Phase 2
Iloprost	Prostacyclin analogue/cAMP modulator	Mechanically ventilated with acute respiratory failure	NCT06319274	Pär Johansson Rigshospitalet/Phase 2
Solnatide (AP301)	ENaC activator	Mechanically ventilated ARDS	EudraCT 2017–003855‐47	Apeptico Forschung und Entwicklung GmbH/Phase 2b
Exoflo	Bone marrow mesenchymal stem cell‐derived extracellular vesicles	Moderate–severe ARDS	NCT05354141	Direct Biologics LLC/Phase 3

**TABLE 2:** Potential ARDS therapeutic targets/agents: Effects on endothelial cells and extracellular vesicle‐mediated mechanisms.

Therapeutic target/agents	Effects on endothelial cells (ECs)	Extracellular vesicle (EV)‐related mechanisms	References
In Clinical Trials			
Extracellular NAMPT (eNAMPT)/ALT‐100 mAb	eNAMPT causes lung EC dysfunction EC‐specific NAMPT‐deficient mice are protected against ALI eNAMPT targeting by ALT‐100 reduces EC permeability and inflammation	eNAMPT is released within EVs NAMPT overexpression induces EV release	Quijada et al. (2021); Yeung et al. (2025); Chong et al. (2022)
TLR4/Paridiprubart	TLR4 ligands cause lung EC dysfunction TLR4 inhibition reduces EC permeability, inflammation, and apoptosis	TLR4 activation triggers EV release EVs activate TLR4 signaling	Yuan et al. (2024); Song et al. (2024, 202); Kim et al. (2022); Yaker et al. (2022)
C5a/Vilobelimab	C5a signaling mediates endothelial dysfunction C5a inhibition reduces pulmonary edema in mice	C5a triggers EV release	Müller‐Redetzky et al. (2020); Wang, Wang, et al. (2021); Song et al. (2018); Karasu et al. (2020); Huang et al. (2015)
VEGF/Bevacizumab	VEGF induces lung endothelial permeability Bevacizumab reduces pulmonary edema	EVs carrying VEGF exhibit endothelial barrier–disruptive properties	Shimizua et al. (2015); Watanabe et al. (2009); Zhao et al. (2018)
Syk/Fostamatinib	Syk promotes lung endothelial barrier disruption Syk inhibition protects against EC dysfunction and lung injury	Syk inhibition suppresses EV release	Shadab et al. (2023); Noomuna et al. (2020); Lhermusier et al. (2011)
p38:MK2/GEn‐1124	Gen‐1124 inhibits lung endothelial permeability	EVs activate p38 signaling	(Tulapurkar et al. 2024; Wang, Lin, et al. 2025)
Neutrophil elastase/Sivelestat	Neutrophil elastase increases EC permeability Endothelial injury and EC glycocalyx degradation are attenuated by sivelestat	EVs are carriers of active neutrophil elastase Neutrophil elastase‐rich EVs promote extracellular matrix degradation	Genschmer et al. (2019); Ushakumari et al. (2022); Suzuki et al. (2019)
IL‐1R/Anakinra	IL‐1β mediates lung endothelial permeability Anakinra combined with an IL‐6R monoclonal antibody inhibits lung endothelial permeability and inflammation	IL‐1β triggers the release of EVs EVs are carriers of IL‐1β	Ganter et al. (2008); Colás‐Algora et al. (2023); Kato et al. (2014); MacKenzie et al. (2001); Wang et al. (2011); Vats et al. (2020)
Tie2/AV‐001	Ang‐1/Tie2 promotes EC barrier integrity Ang‐2 increases permeability Ang‐1 mimetics reduce vascular leakage	EVs are carriers of Ang‐2 and Ang‐1	Gutbier, Neuhauß, et al. (2018); Lask et al. (2022); Zhang, Yang, et al. (2025); Xie et al. (2020)
CD14/IC14	Blocking CD14 protects against LPS‐induced pulmonary edema and EC inflammation	CD14+ EVs are elevated in BAL from patients with sepsis‐related ARDS and poor outcomes	Mahida et al. (2022); Tasaka et al. (2012)
cAMP, prostacyclin/Iloprost	Iloprost protects against lung EC dysfunction	cAMP modulators reduce the production of inflammatory EVs	Birukova et al. (2012); Gąsecka et al. (2021); Coenen et al. (2021)
ENaC/Solnatide	Solnatide inhibits EC hyperpermeability	EVs carry ENaC EVs activate ENaC	Eaton et al. (2025); Hayama et al. (2024); Al‐Humiari et al. (2024)
Mesenchymal Stem Cell (MSC)‐derived EVs/ExoFlo	MSC‐EVs reduce EC dysfunction	MSC‐EVs donate healthy mitochondria to injured EC	Silva et al. (2021)
Preclinical phase			
Sphingosine‐1‐phosphate receptor signaling/S1P analogues (Tysiponate, Fingolimod)	S1P analogues targeting S1PR1 promote lung endothelial barrier integrity	EVs carry S1P EVs mediate cellular responses through S1PR signaling S1P analogues alter EV production and properties	McVey et al. (2021); Wang, Letsiou, et al. (2022); Natarajan et al. (2013); Wang et al. (2014); Deng et al. (2019); Sáenz‐Cuesta et al. (2018)
HMGB1	HMGB1 mediates EC injury	HMGB1 is packaged in EVs	Zhao et al. (2021); Larsson et al. (2025); Wang, Jin, et al. (2021)
TRPV4	TRPV4 signaling mediates lung EC barrier disruption and inflammation	EVs activate TRPV4 signaling	Sonkusare and Laubach (2022); Li, Gao, et al. (2021)
Cell‐free hemoglobin (CFH)	CFH causes lung EC dysfunction	CFH is packaged in EVs	Tomasek et al. (2021); Conger et al. (2023); Pat et al. (2022)

### 3.1 Therapeutics Under Clinical Development

Using ClinicalTrials.gov as the primary source, we reviewed ongoing clinical studies related to ARDS (as of November 2025). For studies involving pharmaceutical interventions, we examined the existing published literature to identify agents with endothelial protective properties (Tables 1 and 2).

#### 3.1.1 eNAMPT—ALT‐100

Nicotinamide phosphoribosyltransferase (NAMPT) has emerged as a novel therapeutic target in ARDS. Intracellularly, NAMPT regulates nicotinamide adenine dinucleotide (NAD) biosynthesis, whereas its extracellular form (eNAMPT) functions as a damage‐associated molecular pattern (DAMP) that activates Toll‐like receptor 4 (TLR4) (Camp et al. 2015). eNAMPT levels are elevated in preclinical ALI animal models and ARDS patients (Camp et al. 2015; Ye et al. 2005; Lynn et al. 2023). Endothelial‐specific NAMPT‐deficient mice are protected against ALI, whereas eNAMPT causes lung EC barrier disruption and induces pro‐inflammatory signaling (Camp et al. 2015; Quijada et al. 2021), findings that provide strong evidence about the role of eNAMPT in regulating lung EC dysfunction. Circulating extracellular NAMPT‐mediated responses can be neutralized by ALT‐100, a monoclonal antibody that was recently developed to mitigate eNAMPT's deleterious effects. ALT‐100 has been effective in reducing lung EC barrier disruption and inflammation in vitro and has beneficial effects in multiple animal models of ALI in vivo (Quijada et al. 2021; Bermudez et al. 2022). The ALT‐100 antibody is currently undergoing phase 2 clinical trials to evaluate its efficacy and safety in individuals with moderate to severe ARDS (NCT05938036). Interestingly, recent studies have shown that eNAMPT is released within EVs (Yeung et al. 2025; Chong et al. 2022), and that FK866, a NAMPT inhibitor, can block the effects of EV‐associated eNAMPT on target cells (Yeung et al. 2025). These findings raise several important questions: Is eNAMPT released within EVs during ARDS? If so, does it contribute to EV‐mediated EC injury? Can ALT‐100 effectively target circulating EVs and their eNAMPT content? In addition, NAMPT has been implicated in the upstream regulation of EV production, as overexpression of NAMPT promotes exosome release in chondrosarcoma cells that polarize macrophages to the M2 phenotype (Song et al. 2024). Future studies are needed to evaluate whether targeting eNAMPT suppresses the release of injurious EVs and/or protects against endothelial dysfunction by diminishing EV‐mediated signaling.

#### 3.1.2 TLR4—Paridiprubart

The efficacy of directly blocking TLR4 signaling is being evaluated in an ongoing phase 2 clinical trial (NCT06701669) using paridiprubart (NI‐0101), a monoclonal antibody against TLR4 (Monnet et al. 2017). TLR4 is activated by a variety of ALI‐related inflammatory insults and results in inflammation and increased EC permeability (Song et al. 1868). Blocking TLR4 represents a promising strategy to reduce lung injury by targeting endothelial permeability, activation, and apoptosis (Kim et al. 2022; Wu et al. 2020; Wang, Wu, et al. 2021). Emerging evidence suggests that TLR4 signaling may be linked to EV‐mediated intercellular communication and inflammatory responses. In a recent study, EVs from PAI‐1 stimulated EC were shown to activate TLR4 and the downstream JAK3/STAT3/IRF‐1 signaling pathway, leading to ALI (Yuan et al. 2024). Moreover, substantial evidence indicates that TLR4 agonists, such as LPS, stimulate the release of pro‐inflammatory EVs (Letsiou and Bauer 2018; Yaker et al. 2022). Taken together, these findings suggest that blocking TLR4 with interventions such as paridiprubart may attenuate both the production of harmful EVs and disrupt EV‐mediated pro‐inflammatory and barrier‐disruptive signaling.

#### 3.1.3 C5a—Vilobelimab

The complement component C5a plays a critical role in the pathogenesis of ARDS (Detsika et al. 2024). C5a is a potent anaphylatoxin generated during complement activation that acts as a strong chemoattractant for inflammatory immune cells and as a mediator of endothelial dysfunction (Detsika et al. 2024; Vahldieck et al. 2024). C5a levels are increased in patients with pneumococcal pneumonia (Müller‐Redetzky et al. 2020) and in patients with ARDS due to COVID‐19 (Carvelli et al. 2020). In a murine model of sepsis induced by *Streptococcus pneumoniae* and mechanical ventilation, neutralization of C5a using the anti‐C5a l‐aptamer NOX‐D19 led to reduced pulmonary permeability and decreased disease severity (Müller‐Redetzky et al. 2020). Similarly, C5a inhibition by W‐54011, a C5a receptor antagonist, reduced LPS‐induced pulmonary edema and lung inflammation in LPS‐treated rats (Wang, Wang, et al. 2021). Blocking the C5a‐C5aR1 axis further inhibited influenza‐induced endothelial activation and damage, and ameliorated ALI in mice (Song et al. 2018). Vilobelimab, a first‐in‐class monoclonal antibody that selectively targets complement component C5a, was evaluated in a randomized, double‐blind, placebo‐controlled Phase 3 trial (PANAMO) for its efficacy in improving survival among critically ill, mechanically ventilated COVID‐19 patients (Vlaar et al. 2022). When administered in addition to standard of care, vilobelimab significantly reduced 28‐day and 60‐day all‐cause mortality compared to placebo, particularly in patients with severe ARDS and impaired renal function (Vlaar et al. 2022). These promising results led to Emergency Use Authorization by the FDA for vilobelimab in this patient population. Currently, vilobelimab is being investigated in an ongoing Phase 2 clinical trial (NCT06701682) to assess its safety and efficacy in hospitalized patients diagnosed with ARDS of various etiologies. In parallel, STSA‐1002, a monoclonal anti‐C5a antibody, is being tested for its safety and efficacy in ARDS patients in a Phase 3 clinical trial (NCT07208591). Interestingly, C5a has been recognized as a stimulus for EV production. C5a triggers the release of pro‐inflammatory or pro‐coagulant EVs from neutrophils (Karasu et al. 2020; Huang et al. 2015). On the basis of these findings, C5a may contribute to ARDS upstream by promoting the release of inflammatory EVs that disrupt endothelial barrier function. Accordingly, C5a‐targeted antibodies may provide benefit by suppressing injurious EV production and limiting downstream endothelial responses to EV‐mediated inflammatory signaling.

#### 3.1.4 VEGF—Bevacizumab

Vascular endothelial growth factor (VEGF) is a pluripotent growth factor that regulates a variety of cellular processes, including angiogenesis, cell proliferation, and permeability. In ARDS, VEGF contributes to vascular endothelial permeability (Shimizua et al. 2015; Watanabe et al. 2009), whereas VEGF plasma levels correlate with higher mortality in ARDS patients (Luo et al. 2025). Currently, an ongoing phase 2 clinical trial is evaluating the efficacy of bevacizumab, a human monoclonal antibody against VEGF, for the treatment of ARDS patients (NCT06701656). Multiple lines of evidence suggest that VEGF is packaged into EVs (Ateeq et al. 2024). Glioblastoma‐derived EVs carry VEGF‐A and induce permeability of the blood–brain barrier (Zhao et al. 2018), suggesting that EV‐associated VEGF could contribute to lung EC barrier disruption during ALI. Notably, EV‐VEGF–mediated effects are not neutralized by bevacizumab, likely because VEGF is sequestered within EVs and therefore inaccessible to the antibody (Ko et al. 2019). This suggests a potential limitation of anti‐VEGF antibody strategies in ARDS if a substantial fraction of extracellular VEGF is packaged within EVs. Critical unresolved questions include determining the role of EV‐associated VEGF in lung endothelial dysfunction and whether bevacizumab modulates its downstream responses in ARDS.

#### 3.1.5 Syk—Fostamatinib

Another potential therapeutic target in ARDS is the non‐receptor tyrosine kinase Syk (Spleen tyrosine kinase). Syk plays a well‐established role in immune regulation and platelet activation (Fischer 2024; Cooper et al. 2023), and emerging evidence highlights a significant link between Syk signaling and the regulation of lung endothelial permeability (Fischer 2024; Shadab et al. 2023). Multiple Syk inhibitors have been developed and tested clinically for a range of indications, including autoimmune diseases, hematological cancers, and COVID‐19 (Cooper et al. 2023). Among these, fostamatinib was approved in 2018 by the US FDA to treat chronic immune thrombocytopenic purpura in adult patients. Fostamatinib is currently under evaluation for the treatment of ARDS in a Phase 2 clinical trial (NCT06564207), although previous studies in COVID‐19 patients were discontinued because of a lack of demonstrated clinical benefit. Although the direct effects of fostamatinib on EVs are not yet known, recent studies demonstrate that R406, its active metabolite, reduces EV production from activated RBCs and platelets (Noomuna et al. 2020; Lhermusier et al. 2011). If fostamatinib similarly inhibits the release of EVs, this could confer important downstream protective effects. Activated neutrophils, platelets, and RBCs are major sources of endothelial‐injuring EVs, and Syk inhibition reduces activation of all these cell types (Noomuna et al. 2020; Zhu et al. 2023; Clarke et al. 2018). Thus, upstream suppression of injurious EV production is a plausible mechanism by which Syk inhibition promotes endothelial barrier protection, potentially contributing to the barrier‐protective effects of Syk inhibitors in inflammatory lung injury.

#### 3.1.6 p38:MK2—GEn‐1124

GEn‐1124, a compound with anti‐inflammatory and endothelial stabilizing properties, is currently being tested for efficacy and tolerability in a phase 2 clinical trial for ARDS treatment (NCT05795465) (Tulapurkar et al. 2024). Regarding its mechanism of action, GEn‐1124 destabilizes the p38:MK2 (mitogen‐activated protein kinase‐activated protein kinase) complex and inhibits thrombin‐induced EC permeability and protects against LPS or viral‐induced murine ALI (Tulapurkar et al. 2024). It remains unknown whether EVs produced in ARDS can activate p38:MK2 signaling pathways in relevant cells that could be targeted by GEn‐1124. Evidence from other disease models indicates that EVs from PM2.5‐treated microglia can activate p38 signaling in neurons through the delivery of miR‐34a‐5p (Wang, Lin, et al. 2025). This miRNA has been implicated in EC dysfunction and inflammation in the lungs (Shah et al. 2019). Accordingly, an attractive approach would be to determine whether EVs generated during ARDS carry cargo that activates p38 signaling in lung EC and whether such EV‐mediated signaling could be therapeutically attenuated by inhibition of p38 signaling with agents such as GEn‐1124.

#### 3.1.7 NE—Sivelestat

Sivelestat sodium is a NE inhibitor being tested for its efficacy to inhibit the development of ARDS in septic patients (NCT04973670) (Ma et al. 2023) as well as its ability to treat established ARDS (NCT06387823). Sivelestat's protective effects have been demonstrated in several preclinical models of lung injury (Ma et al. 2023). Pulmonary endothelial injury and EC glycocalyx degradation are attenuated in sivelestat‐treated endotoxemic mice (Suzuki et al. 2019). Mechanistically, NE promotes endothelial dysfunction by activating protease‐activated receptor‐2 (PAR‐2), leading to disruption of inter‐endothelial junctions through VE‐cadherin cleavage and a consequent increase in endothelial permeability. Inhibition of NE mitigates these effects, thereby reducing vascular injury and leukocyte infiltration (Ushakumari et al. 2022). Interestingly, as discussed in the previous section, NE‐enriched EVs further contribute to extracellular matrix degradation and progression of lung disease (Genschmer et al. 2019). Consistent with this emerging role, a recent preprint reports that sivelestat suppresses cellular responses induced by EV‐associated NE, highlighting an additional mechanism by which elastase inhibition may confer vascular and tissue protection (Oshins et al. 2024). All together, these findings raise the possibility that sivelestat may act directly on EV‐associated NE to attenuate EV‐mediated endothelial dysfunction. Further investigation is warranted to establish whether targeting EV‐bound NE constitutes a significant and therapeutically relevant strategy for endothelial protection in ARDS.

#### 3.1.8 IL‐1R—Anakinra

ARDS is marked by excessive production of pro‐inflammatory cytokines that play a central role in driving lung injury. Among these, interleukin‐1 (IL‐1), including both IL‐1α and IL‐1β, has been well documented for its potent inflammatory effects in the lungs. In particular, IL‐1β is a well‐established ARDS mediator. It is produced by various lung cells and immune cells and is released upon inflammasome activation. It exerts a wide range of injurious effects, including the disruption of lung endothelial and epithelial barrier integrity, leading to increased permeability (Ganter et al. 2008). Interleukin‐1 receptor antagonist (IL‐1Ra) is a naturally occurring 25‐kDa plasma protein that competes with IL‐1α and IL‐1β for binding to the type I IL‐1 receptor, effectively blocking downstream inflammatory signaling. A recombinant form of IL‐1Ra antagonist, known as anakinra, is a 17‐kDa nonglycosylated protein that is FDA‐approved for the treatment of several inflammatory and autoimmune disorders, including rheumatoid arthritis. Beyond these established uses, anakinra has been studied for its therapeutic potential in sepsis and COVID‐19 (Shakoory et al. 2016; Iglesias‐Julián et al. 2020), whereas another clinical trial was designed to evaluate its efficacy in patients with ARDS (NCT05914454). Anakinra combined with an IL‐6R monoclonal antibody effectively reduces cytokine‐induced lung endothelial permeability and inflammatory signaling (Colás‐Algora et al. 2023). There is evidence that anakinra's protective effects may also involve EV‐mediated mechanisms. IL‐1β stimulates the production of EVs (Kato et al. 2014), and it is a well‐recognized component of EVs from cells stimulated with inflammatory signals (MacKenzie et al. 2001; Wang et al. 2011).

An earlier study showed that LPS‐treated monocytes produce EVs‐carrying IL‐1β, causing EC activation, a process that was inhibited after blocking the IL‐1R receptor in EC (Wang et al. 2011). In dengue virus infections, inflammasome activation in platelets results in the release of IL‐1β‐rich EVs, which mediate EC barrier disruption (Hottz et al. 2013). Similarly, in sickle cell disease, inflammasome‐dependent shedding of IL‐1β and caspase‐1‐containing platelet‐derived EVs promotes lung vasoocclusion, a process that is blocked by treatment with recombinant IL‐Ra (Vats et al. 2020). Taken together, inhibition of IL‐1 receptor signaling may act at multiple levels to limit EV‐mediated endothelial injury, including upstream suppression of EV release, potential neutralization of IL‐1β contained within EVs, and downstream attenuation of EV‐driven inflammatory signaling in EC. However, direct experimental evidence supporting these mechanisms in the context of ARDS remains limited, highlighting the need for targeted studies to define how IL‐1–directed therapies intersect with EV biology.

#### 3.1.9 Tie2/Ang—AV‐001

The Tie2 receptor and its angiopoietin ligands constitute a critical signaling axis involved in regulating vascular stability during sepsis and ARDS (Sack et al. 2020). Tie2 is highly expressed in ECs, and the Tie2/Ang pathway is a critical modulator of EC health. Although angiopoietin‐1 (Ang‐1) signaling is EC barrier protective, angiopoietin‐2 (Ang‐2) conversely mediates endothelial permeability and inflammatory signaling (Gutbier, Neuhauß, et al. 2018). Ang‐2 levels are increased in patients with ARDS and are associated with disease severity and prognosis (Rosenberger et al. 2023). AV‐001 is a synthetic Ang‐1 mimetic peptide that is a promising therapeutic to target the Tie2 pathway (Culmone et al. 2022). AV‐001 is being investigated in a phase 2a clinical trial in hospitalized patients with pneumonia requiring supplemental oxygen therapy (NCT05123755). Other similar therapeutics are being developed in parallel. For example, vasculotide is an Ang‐1 mimetic, which is effective in reducing pulmonary hyperpermeability in murine models of severe pneumonia and sepsis (Lask et al. 2022). Our understanding of EV‐mediated mechanisms in relation to the Tie2 signaling pathway remains limited; however, emerging evidence suggests that EVs can carry angiopoietins and transfer them to target cells (Zhang, Yang, et al. 2025; Xie et al. 2020). In a recent study, pericyte‐derived EVs carrying Ang‐1 reduced EC permeability to LPS in vitro and improved vascular hyperpermeability in vivo using the CLP model (Zhang, Yang, et al. 2025). Ang‐2 carried by EVs from cancer cells can be taken up by EC (HUVEC) to alter their angiogenesis (Xie et al. 2020). Notably, in the context of COVID‐19, elevated levels of Ang‐2 were associated with activation of coagulation and release of pro‐coagulant EVs (Barbosa et al. 2024), highlighting a potential intersection between Tie2 signaling and EV‐mediated pathophysiology. It remains to be determined (1) whether EVs carry Ang‐2 during ARDS to promote lung EC dysfunction, and (2) if compounds such as AV‐001 and vasculotide exert protective effects on ECs by inhibiting Ang‐2‐associated EV signaling.

#### 3.1.10 CD‐14—IC14

Anti‐CD14 treatment with a recombinant chimeric monoclonal antibody (IC14) is being evaluated in a phase 2 trial for safety and efficacy in hospitalized patients with ARDS (NCT06513949). The cluster of differentiation 14 (CD14) antigen facilitates the binding of pathogen‐associated molecular patterns (PAMPs) and DAMPs to TLRs. Blocking CD14 in LPS‐treated mice attenuates ALI, as evidenced by reduced pulmonary edema and decreased neutrophil infiltration (Tasaka et al. 2012). CD14 can function as a co‐receptor or scavenger for LPS, and earlier studies have shown that although ECs express CD14 at significantly lower levels compared to monocytes, its expression is upregulated following LPS stimulation (Jersmann et al. 2001). Treatment with anti‐CD14 antibodies reduces endotoxin‐induced IL‐6 and E‐selectin expression in ECs (von Asmuth et al. 1993), suggesting that CD14 plays a critical role in mediating the inflammatory response to LPS and may represent a potential therapeutic target for modulating endothelial activation in ARDS. Notably, CD14+/CD81+ – EV levels are significantly elevated in BAL of sepsis‐related ARDS patients who died during the first 30 days following ICU admission, compared to survivors (Mahida et al. 2022). The functional role of these EVs remains to be defined, particularly their capacity to target the lung endothelium and the potential impact of CD14 antibody treatment on these processes.

#### 3.1.11 cAMP Modulators—Iloprost

Clinical and preclinical trials have evaluated the potential of iloprost to mitigate endothelial dysfunction and reduce lung injury. Iloprost is a prostacyclin analogue with endothelial protective properties. Earlier studies have demonstrated that iloprost attenuates endothelial permeability and inflammatory signaling induced by LPS through a mechanism that at least partially involves elevation of intracellular cAMP levels (Birukova et al. 2012). Clinically, inhaled iloprost improved oxygenation in severe COVID‐19 patients (Serbanescu‐Kele Apor de Zalán et al. 2022); however, in ARDS patients of various etiologies, it failed to provide significant benefits in terms of oxygenation, mortality, duration of mechanical ventilation, or length of ICU stay (Haeberle et al. 2023). Currently, intravenous administration of iloprost is being evaluated for the treatment of mechanically ventilated patients with acute respiratory failure (NCT06319274). Given the potent EC protective properties of iloprost, this route of administration may offer a more consistent therapeutic effect by ensuring targeted drug delivery to the vasculature. Multiple lines of evidence suggest that iloprost targets EV‐mediated mechanisms. EVs from TNF‐α–stimulated monocyte–platelet aggregates exert pro‐inflammatory effects on ECs; however, when iloprost is present, activation of these monocyte‐platelet aggregates produces EVs with distinct, less inflammatory proteomes (Oggero et al. 2021). Similarly, patients with pulmonary arterial hypertension (PAH) treated with iloprost exhibited less platelet activation and reduced circulating levels of platelet and leukocyte EVs (Gąsecka et al. 2021). Circulating endothelial EVs represent a marker of endothelial injury in systemic sclerosis patients and are reduced in patients receiving iloprost (Argentino et al. 2024). Therefore, in the context of ARDS, iloprost is likely to influence both the quantity and the biological activity of released EVs, as well as their downstream effects on the pulmonary endothelium. Several other compounds that alter cAMP levels are currently in trials or approved for various indications, such as ensifentrine, which is FDA‐approved to treat COPD (Almuntashiri et al. 2025). Ensifentrine is a dual phosphodiesterase (PDE) 3/4 inhibitor that directly causes cAMP elevation. We recently demonstrated that it protects against lung endothelial barrier disruption and reduces inflammation in lung endothelial and epithelial cells after MRSA exposure (Al Matni et al. 2024). Our unpublished data further demonstrate that pretreatment with ensifentrine in injured alveolar epithelial cells attenuates both the release of EVs and their barrier‐disruptive effects (Letsiou et al. 2024). Consistent with these findings, cilostazol, a PDE3 inhibitor and cAMP inducer, reduces platelet activation and subsequent release of procoagulant EVs under inflammatory conditions (Coenen et al. 2021). Interestingly, treatment of lung microvascular ECs with PDE inhibitors under normal conditions results in the production of cAMP‐rich EVs that improved outcomes in a hypoxia‐induced pulmonary hypertension rat model (Sayner et al. 2019; Bhadra et al. 2026). Taken together, these findings suggest that cAMP modulators exert direct protective effects on the endothelial barrier while also modulating the release and downstream functions of EVs released from various cell types.

#### 3.1.12 ENaC—Solnatide

Extensive research has established the epithelial sodium channel (ENaC) as a promising therapeutic target in the treatment of ARDS. ENaC is a non‐voltage‐gated, amiloride‐sensitive ion channel that regulates sodium ion transport. It plays a crucial role in regulating sodium homeostasis and fluid balance (Chen et al. 2023), and therefore it is an important mediator of alveolar fluid clearance, which is severely impaired in ARDS. In addition to its well‐established role in alveolar epithelial cells, ENaC is now recognized to be expressed in lung EC, where it plays a key role in modulating barrier function and inflammatory signaling (Eaton et al. 2025). In mice treated with LPS, pulmonary endothelial permeability is significantly increased in those with a conditional, endothelial‐specific knockout of ENaC‐α compared to control mice (Sternak et al. 2018). TIP (thrombin‐interacting peptide) activates ENaC and protects against lung endothelial permeability and oxidative stress caused by various inflammatory insults in vitro and in vivo (Eaton et al. 2025). Several clinical trials have tested the safety and efficacy of TIP (AP301 or Solnatide) to treat ARDS (Eaton et al. 2025). In a phase 2a clinical trial, no overall therapeutic efficacy of AP301 was observed in patients with a lower level of clinical severity (SOFA scores ≤ 10). However, inhaled AP301 reduced the extravascular lung water index (EVLWI) in more severely ill patients with SOFA scores ≥ 11 (Krenn et al. 2017). The TIP peptide (Solnatide) has also been evaluated in a multicenter, double‐blind, dose‐escalation phase 2b clinical trial in both COVID‐related and non‐COVID ARDS patients undergoing invasive mechanical ventilation (NCT03567577) (Schmid et al. 2021). Although some studies have shown that ENaC‐γ subunit can be transported via EVs (Hayama et al. 2024), and others suggest that EVs can activate ENaC (Al‐Humiari et al. 2024), there is currently no clear evidence linking the TIP peptide's mechanism of action to EV‐mediated pathways. However, given TIP's protective effects in both alveolar epithelial and lung ECs, it would be important to determine whether it modulates the release, composition, or activity of EVs derived from these cells during inflammation. For example, we have previously shown that alveolar epithelial cells release high levels of inflammatory EVs in response to pneumolysin (Letsiou, Teixeira Alves, et al. 2021; Letsiou et al. 2023), a toxin for which TIP has been shown by others to confer protection to lung EC (Czikora et al. 2017). Could TIP act upstream to inhibit the release of injurious EVs from pneumolysin‐stimulated cells, or does it instead protect lung EC downstream by increasing resistance to pneumolysin‐associated EV‐mediated injury? These are compelling questions that warrant further investigation.

#### 3.1.13 Mesenchymal Stem Cell‐Derived EVs—ExoFlo

Although this review emphasizes the injurious effects of a wide variety of EVs, a substantial body of evidence highlights the potent cytoprotective properties of mesenchymal stem cell‐derived EVs (MSC‐EVs), which are considered strong therapeutic candidates for a range of conditions, including ARDS (Abraham and Krasnodembskaya 2019). A phase 3 clinical trial is evaluating the safety and efficacy of intravenous (IV) administration of bone marrow MSC‐EVs (ExoFlo) for the treatment of hospitalized patients with moderate‐to‐severe ARDS (NCT05354141). Preclinical studies have demonstrated that MSC‐EVs can alleviate the increased permeability of lung endothelium induced by either LPS or plasma from ARDS patients with a hypo‐inflammatory phenotype (Silva et al. 2021). These MSC‐EVs contain functional mitochondria that can be transferred to injured EC to restore their mitochondrial function (Silva et al. 2021). Additionally, MSC‐EVs reduce IL‐8 release from LPS‐treated ECs, although it remains unclear whether this effect is dependent on mitochondrial transfer (Silva et al. 2021).

### 3.2 Emerging Therapeutics in Pre‐Clinical Development

A growing number of promising therapeutic targets for ARDS are currently under preclinical investigation. In this section, we highlight select candidates, focusing on their roles in EC function and their interplay with EVs.

#### 3.2.1 S1P/S1PR

Sphingosine‐1‐phosphate (S1P) is a naturally occurring bioactive sphingolipid that exerts its effects both extracellularly through its G protein‐coupled receptors, S1P1–5, and intracellularly by interacting with various molecular targets (Natarajan et al. 2013). We and others have demonstrated that S1P is an important regulator of vascular EC barrier in vitro and in vivo (Natarajan et al. 2013). In general, activation of S1PR1 is associated with endothelial barrier enhancement; S1PR3 mediates S1P‐induced barrier‐disruptive and pro‐inflammatory signaling, and S1PR2 exhibits context‐dependent protective or disruptive effects (Singleton et al. 2005; Ha et al. 2025; Wu et al. 2023). The S1P–S1PR signaling axis is implicated in various disease processes, which has stimulated the development of numerous S1P analogues as potential therapeutic agents. These include FTY‐720 (Fingolimod), ozanimod, and ponesimod, which are all FDA‐approved for multiple sclerosis. We have extensively studied the effects of various S1P analogues on EC function and tested their efficacy to treat ALI in preclinical models (Camp et al. 2009). Among these, the (S)‐FTY720‐phosphonate analogue, Tysiponate or Tys, has demonstrated the ability to enhance EC barrier function both in vitro and in vivo against a range of ALI triggers, including LPS, bleomycin, and MRSA (Wang, Letsiou, et al. 2022; Camp et al. 2009; Wang et al. 2014). Unlike other S1P analogues, Tys does not appear to cause immunosuppression and uniquely preserves S1PR1 expression, a key feature that contributes to its role in maintaining vascular barrier integrity (Wang et al. 2014). There is growing evidence of a dynamic interplay between EVs and S1P signaling. EVs can serve as a source of extracellular S1P (McVey et al. 2021), contributing to the local signaling environment, and several studies show that EVs activate S1P pathways in recipient cells, influencing cellular responses (Deng et al. 2019). Moreover, S1P itself can regulate EV release; for example, in patients with multiple sclerosis, treatment with the S1P analogue fingolimod alters both the levels and functional properties of circulating EVs (Sáenz‐Cuesta et al. 2018; Zinger et al. 2016). The potential effects of Tys on EV release and function are currently being investigated in our ongoing studies. Mechanistically, S1P promotes cytoskeletal rearrangement in EC, which is a key driver of EV biogenesis (Morel et al. 2011). Our prior studies have demonstrated that the actin related protein 2/3 (Arp 2/3) complex, a regulator of peripheral branched actin polymerization, mediates the EC protective effects induced by S1P (Belvitch et al. 2017). Interestingly, Arp2/3 was recently shown to be involved in EV release (de Poret et al. 2022). Whether EVs induced via S1P‐Arp2/3 signaling exert barrier protective properties remains unknown. Altogether, these findings support a mechanistic link between S1P signaling and EV biology; however, further studies are needed to determine whether therapeutics targeting S1PR pathways modulate EV release, cargo composition, and EV‐mediated functional effects on the lung endothelium in ARDS.

#### 3.2.2 HMGB1

High Mobility Group Box 1 Protein (HMGB1) is a DAMP that plays a central role in the progression of both infectious and sterile inflammatory conditions (Andersson et al. 2020). After extracellular release, HMGB1 binds to receptors such as the Receptor for Advanced Glycation End Products (RAGE) and TLRs, triggering downstream signaling pathways that culminate in NF‐κB activation. This promotes endothelial barrier hyperpermeability in a dose‐ and time‐dependent manner, along with cytoskeletal stress fiber rearrangement and the disruption of junctional proteins like VE‐cadherin and ZO‐1 (Zhong et al. 2020; Zhao et al. 2021). Elevated HMGB1 levels have been observed in COVID‐19 patients and are strongly correlated with disease severity (Wulandari et al. 2023). Moreover, HMGB1 serves as an independent predictor of mortality in ARDS patients in the ICU setting (Tseng et al. 2014). Various compounds, including thrombomodulin (Kudo et al. 2013), calycosin (Chen et al. 2021), and glycyrrhizin (Buder et al. 2022) have shown potential in reducing HMGB1‐mediated inflammation. Emerging evidence indicates that HMGB1 is also closely related to the activation of inflammasomes (Liu et al. 2025; Kim, Park, et al. 2018), which mediates endothelial dysfunction (Liu et al. 2025) and participates in the development of LPS‐induced ALI in murine models (Grailer et al. 2014). Pharmacologic agents such as tetracycline (Peukert et al. 2021), melatonin (Zhang et al. 2016), and erythropoietin (Cao et al. 2020) have demonstrated protective effects by modulating this pathway. Several studies have demonstrated that HMGB1 can be packaged within EVs. In ALI‐relevant studies, EVs carrying HMGB1 were found in BAL of mice exposed to high‐tidal volume ventilation (Larsson et al. 2025). Macrophages stimulated with LPS release HMGB1 via EVs, which subsequently activate the inflammasome and induce pyroptosis in recipient cells (Wang, Jin, et al. 2021). These findings suggest that pharmacologic strategies that directly target the EV‐encapsulated HMGB1 cargo may preserve endothelial barrier integrity and diminish pro‐inflammatory signaling during ARDS.

#### 3.2.3 TRPV4

Transient Receptor Potential Vanilloid 4 (TRPV4) has emerged as a promising therapeutic target in ARDS because of its central role in regulating pulmonary vascular permeability and inflammation (Balakrishna et al. 2014; Morty and Kuebler 2014; Sonkusare and Laubach 2022). TRPV4 is a mechanosensitive, calcium‐permeable ion channel expressed in various cells, including lung endothelial and epithelial cells. Its activation by a range of ALI‐related insults leads to endothelial barrier disruption, pro‐inflammatory signaling, and subsequent pulmonary edema and tissue damage (Villalta and Townsley 2013). However, contrasting evidence suggests that TRPV4 also plays a protective role in maintaining alveolar barrier integrity and preventing edema formation under certain conditions (Weber et al. 2020). Nonetheless, multiple TRPV4 inhibitors have been developed and evaluated across various disease models, with their therapeutic efficacy showing considerable variability. Among these, GSK2193874 suppresses inflammatory responses in mechanically stretched human epithelial cells and reduces pulmonary barrier permeability and cytokine release in a murine model of VILI (Pairet et al. 2018). In contrast, GSK2798745, another TRPV4 inhibitor, did not significantly impact critical disease measures in a swine model of chlorine gas‐induced ALI (Vermillion et al. 2024). TRPV4 activation induces calcium influx, a known driver of EV release. Although it remains unclear whether TRPV4 signaling contributes directly to the generation of inflammatory EVs, recent findings show that EVs released from activated platelets can engage TRPV4 in vascular smooth muscle cells, triggering calcium oscillations (Li, Gao, et al. 2021). These observations raise the possibility that EVs produced during ALI may activate TRPV4 signaling in lung EC, and that TRPV4 inhibition could interrupt this pathway.

#### 3.2.4 Cell‐Free Hemoglobin

As discussed in the previous section, a substantial amount of evidence indicates that cell‐free hemoglobin (CFH), when released from damaged erythrocytes, plays a critical role in the development of sepsis and ARDS (Meegan et al. 2021; Shaver et al. 2016). Plasma levels of CFH are increased in ARDS (Janz and Ware 2015), and it is well established that CFH directly causes endothelial barrier disruption and triggers oxidative stress and pro‐inflammatory signaling (Tomasek et al. 2021; Conger et al. 2023). In addition to EC, CFH can activate various other cells such as platelets, neutrophils (Ross et al. 2024; Villagra et al. 2007), and alveolar epithelial cells (Shaver et al. 2016). As summarized in (Meegan et al. 2021), several anti‐hemoglobin therapies, including haptoglobin (hemoglobin scavenger), acetaminophen (hemoprotein reductant), or ascorbate, have shown promise in targeting CFH‐mediated effects in preclinical models; however, further investigation is required to advance these findings toward clinical application. As EVs carrying hemoglobin exert injurious properties (Pat et al. 2022), it would be important to evaluate their levels in ARDS and investigate whether they are involved in lung EC dysfunction. Furthermore, oxidized CFH has been shown to induce mitochondrial dysfunction in lung EC (Riedmann et al. 2025), a process we have previously linked to EV release (Nerlich et al. 2018; Letsiou et al. 2023). Investigating whether CFH stimulates the release of EVs that contribute to endothelial barrier disruption and inflammation could provide valuable insights. Finally, evaluating the impact of CFH‐targeted therapies on EV release, cargo, and biological properties may reveal novel strategies for mitigating CFH‐induced endothelial injury in ARDS.

### 3.3 Limitations and Considerations

In this section, we focused primarily on emerging and clinically advanced therapeutic strategies for ARDS, with an emphasis on proposed mechanisms of action in the lung endothelium and potential efficacy against injurious EV‐mediated signaling. As such, we have not provided a comprehensive discussion of adverse effects, off‐target consequences, or safety liabilities associated with these interventions. Many therapies highlighted are in preclinical or early clinical stages, where safety profiles remain incompletely characterized and may evolve as development progresses. A more detailed evaluation of toxicity, immunosuppression, vascular side effects, and long‐term outcomes will be essential as these approaches advance toward clinical implementation, but they are outside the scope of the current review.

Therapeutic strategies under investigation for ARDS encompass multiple drug modalities, including small molecule agents (inhibitors, agonists, synthetic peptides, etc.) and biologics (monoclonal antibodies, recombinant antagonists, etc.), each with distinct advantages and limitations. Monoclonal antibodies primarily target extracellular molecules and offer high target specificity and vascular accessibility. However, their clinical use may be limited by high cost, complex pharmacokinetics, lack of orally administered options, and the need for precise timing of administration. In contrast, small molecule therapeutics can modulate both extracellular and intracellular signaling pathways, but their utility may be constrained by off‐target effects, poor solubility or bioavailability, short half‐lives, and increased risk of toxicity. With respect to targeting EVs, it remains unclear whether the type of therapy has a decisive impact on efficacy. Broadly, EV‐directed therapeutics aim to: (1) inhibit EV biogenesis and release, (2) prevent EV uptake or interaction with recipient cells, and (3) modify EV cargo. Monoclonal antibodies may be particularly well suited for blocking the interaction of EVs with target cells. In addition, antibodies can indirectly modulate EV biogenesis by interfering with upstream or downstream signaling pathways that regulate EV release. For example, antibody‐mediated targeting of TLR4 could inhibit downstream signaling cascades that promote the release of inflammatory EVs. Small molecule therapeutics, on the other hand, may be more effective at directly inhibiting EV biogenesis and altering EV cargo composition through intracellular mechanisms. Small molecules could also contribute to EV neutralization or uptake inhibition by targeting key molecules/pathways involved in EV‐cell interactions. Future studies will be essential to determine which therapeutic strategy is most effective for modulating EV‐mediated signaling.

The development of effective pharmacologic therapies for ARDS has been hindered in large part by the biological complexity and heterogeneity of the syndrome. ARDS can arise from diverse clinical insults, including viral and bacterial pneumonia, sepsis, and aspiration, which converge on common pathological features such as alveolar–capillary barrier disruption, pulmonary edema, and excessive inflammation, yet they are driven by distinct underlying mechanisms. Beyond etiology, ARDS patients can be stratified into biologically distinct subphenotypes—most notably hyperinflammatory and hypoinflammatory groups—on the basis of biomarker profiles, transcriptomics, and clinical variables (Levine and Calfee 2023; Ma et al. 2025). The hyperinflammatory subphenotype is characterized by elevated levels of circulating inflammatory markers, a higher prevalence of sepsis, and substantially worse outcomes, whereas the hypoinflammatory subphenotype is associated with lower mortality (Levine and Calfee 2023). Importantly, treatment responses in ARDS have been shown to vary by subphenotype, as in the case of simvastatin (Calfee et al. 2018), highlighting the importance of patient stratification in therapeutic evaluation. Given that the extent of EC injury likely differs across ARDS etiologies and subphenotypes (Cusack et al. 2023), these sources of heterogeneity should be considered when assessing the efficacy of investigational therapies. It is also probable that EV abundance, cellular origin, cargo composition, uptake, and endothelial effects vary among ARDS patients on the basis of both etiology and biological subphenotype. Although emerging evidence supports associations between EV characteristics and ARDS severity (Mahida et al. 2022; do Nascimento et al. 2025; Guervilly et al. 2011; Shaver et al. 2017), integration of EV profiling with validated ARDS subphenotyping strategies remains limited. Addressing this gap may provide critical insight into disease heterogeneity and enable more precise targeting of EV‐mediated endothelial dysfunction.

## 4 Conclusion

The lung endothelium is a critical site of injury in ARDS, regardless of whether the syndrome is triggered by direct pulmonary insults or indirect extrapulmonary causes, making it an attractive target for therapeutic intervention. Because of its strategic location and constant exposure to circulating factors, lung ECs are particularly susceptible to inflammatory mediators, including EVs originating from both local pulmonary cells and distant organs (Figure 1). Although significant progress has been made in identifying EVs as both biomarkers and mediators of ALI, their specific impact on the lung endothelial barrier remains incompletely understood. A deeper and more integrated understanding of how EVs from diverse cellular, tissue, and organ sources contribute to endothelial dysfunction—and how emerging therapeutics may modulate these pathways—will be critical for developing targeted strategies to preserve endothelial barrier integrity and mitigate lung injury.

## Author Contributions

M.Y.A.M. drafted sections of the manuscript; P.B. drafted and revised sections of the manuscript; S.M.D. performed major revisions and critically reviewed the manuscript; E.L. conceived the article, drafted and revised the manuscript, and prepared the figures and tables. All authors approved the final manuscript.

## Funding

This work was supported by the American Heart Association (932176/EL/2022), American Lung Association, 1445152 (EL) and National Institutes of Health, R01 HL167518 (SD), R01 HL 170118 (PB).

## Disclosure

Portions of this review were reviewed and edited for grammar and readability using Microsoft Copilot. The authors retain full responsibility for the content, interpretation, and accuracy of the work.

## Conflicts of Interest

The authors declare no conflicts of interest.

## Data Availability

The data that support the findings of this study are available from the corresponding author upon reasonable request.
